# Performance evaluation of six popular short-read simulators

**DOI:** 10.1038/s41437-022-00577-3

**Published:** 2022-12-10

**Authors:** Mark Milhaven, Susanne P. Pfeifer

**Affiliations:** 1 School of Life Sciences, Arizona State University, Tempe, AZ 85281 USA; 2 Center for Evolution and Medicine, Arizona State University, Tempe, AZ 85281 USA

**Keywords:** Genetic variation, Evolutionary genetics, Next-generation sequencing

## Abstract

High-throughput sequencing data enables the comprehensive study of genomes and the variation therein. Essential for the interpretation of this genomic data is a thorough understanding of the computational methods used for processing and analysis. Whereas “gold-standard” empirical datasets exist for this purpose in humans, synthetic (i.e., simulated) sequencing data can offer important insights into the capabilities and limitations of computational pipelines for any arbitrary species and/or study design—yet, the ability of read simulator software to emulate genomic characteristics of empirical datasets remains poorly understood. We here compare the performance of six popular short-read simulators—ART, DWGSIM, InSilicoSeq, Mason, NEAT, and wgsim—and discuss important considerations for selecting suitable models for benchmarking.

## Introduction

High-throughput sequencing has become a cornerstone of biological research, with applications spanning a diverse range of scientific disciplines, including agricultural, comparative, ecological and evolutionary genomics, clinical diagnostics, and personalized medicine. Although researchers can presently sequence the genome of many organisms within days at moderate to low costs (van Nimwegen et al. 2016), the scale and complexity of the produced data raise significant challenges for de novo genome assembly, read mapping, variant calling, and genotyping as well as for the interpretation of the obtained results (see Pfeifer 2017 for a discussion of the challenges and guidelines in short-read sequencing).

In concert with the recent advances in high-throughput sequencing technology, many software tools have been developed for the computational processing of genomic sequencing data, each with their own distinct errors and biases. In fact, systematic comparisons of computational pipelines across multiple high-throughput sequencing platforms indicated high divergence and low concordance among the identified variants (O’Rawe et al. 2013; Pirooznia et al. 2014; Hwang et al. 2015; Chen et al. 2019; Kumaran et al. 2019; Krishnan et al. 2021; Barbitoff et al. 2022). Such differences in performance are particularly problematic for the identification of spontaneous (de novo) mutations as well as rare variants, with differences in pipeline design leading to several-fold variation in estimated mutation rates (Pfeifer 2021; Bergeron et al. 2022) as well as high rates of missed variants (Peng et al. 2013).

Given the crucial impact of computational analysis pipelines on the reliability and robustness of results, careful benchmarking is required both to assess the performance of existing and newly designed computational genomic pipelines as well as to quantify their sensitivity and specificity for any given study. In humans, a handful of high-quality empirical datasets exist for this purpose (such as the “gold-standard” Genome In A Bottle dataset maintained by the U.S. National Institute of Standards and Technology; Zook et al. 2014), but similar datasets remain absent for most non-model organisms. For these species, synthetic (i.e., simulated) sequence data provide an alternative means to guide the development and validation of computational pipelines in silico. In contrast to empirical data, simulations allow for the implementation of controlled scenarios with parameters of arbitrary complexity for which the “ground truth” is known a priori. Importantly, knowledge of this “ground truth” does not only enable benchmarking and performance comparisons, it also allows researchers to distinguish between distinct (often hypothetical) biological scenarios and to evaluate the capabilities and limitations of particular study design choices (for a discussion on the topic, see the recommendations for improving statistical inference in population genomics by Johri et al. 2022). In addition, simulations can guide future experimental designs and aid parameter optimization by studying the potential impact of technological factors (such as sample size, sequencing coverage, as well as quality, continuity, and completeness of available reference assemblies) on downstream analyses (Stephens et al. 2016).

Although synthetic data is clearly highly valuable, it is important that it faithfully resembles both platform-specific features of the sequencing data (such as read type and length, fragment size distribution, rates and patterns of sequencing error, quality score distribution, and, if applicable, PCR amplification bias due to differential efficiencies of primers), as well as the biological/genomic characteristics of the organism, studied (such as rates of substitution, insertion, and deletion, GC-content, etc.). Over the past years, several software packages have been developed to simulate realistic high-throughput genomic datasets with and without the ability to spike in known variants (e.g., Ewing et al. 2015), the majority of which focused on emulating data generated by Illumina sequencing—one (if not currently the most) widely-used sequencing technology in research applications (see reviews by Escalona et al. 2016, Zhao et al. 2017, and Alosaimi et al. 2020). In general, these methods use either pre-defined “basic” models or “advanced” parameterized custom models designed to mimic the genomic characteristics of the empirical sequence dataset at hand—however, as previously noted by Escalona et al. (2016), these tools “have largely not been benchmarked or validated” independently.

Here, we compare six short-read simulators—ART (Huang et al. 2012), DWGSIM (Homer 2022), InSilicoSeq (Gourlé et al. 2019), Mason (Holtgrewe 2010), NEAT (Stephens et al. 2016), and wgsim (Li et al. 2009) (Table 1), selected based on their popularity within our scientific community—to assess their ability to accurately mimic characteristic features (namely, genomic coverage, distribution of fragment lengths, quality scores, and systematic errors, as well as GC-coverage bias) of real data obtained from Illumina sequencing for which error models have been well-characterized.

**Table 1:** Characteristics of the short-read simulators included in this study.

Characteristic	ART	DWGSIM	ISS	Mason	NEAT	wgsim
Read length	36 bp (GenomeAnalyzer I) 44 bp (GenomeAnalyzer I) 50 bp (GenomeAnalyzer II) 50 bp (MiniSeq) 75 bp (GenomeAnalyzer II) 75 bp (NextSeq 500) 100 bp (HiSeq 1000/2000) 125 bp (HiSeq 2500) 150 bp (HiSeqX) 250 bp (MiSeq)	variable	125 bp (Basic) 126 bp (HiSeq) 151 bp (NovaSeq) 301 bp (MiSeq)	variable	variable	variable
Read type	single-end (SE) paired-end (PE) mate-pair (MP)	single-end (SE) paired-end (PE) mate-pair (MP)	paired-end (PE)	single-end (SE) paired-end (PE) mate-pair (MP)	single-end (SE) paired-end (PE)	single-end (SE)
Input	# reads	# read pairs	# reads	# read pairs	coverage	# read pairs
Error model (default)	Dependent on selected Illumina platform	Illumina	Basic HiSeq NovaSeq MiSeq	Illumina	Illumina	Illumina
Error model (customizable)	Yes	Yes	Yes	Yes	Yes	Yes
Provides scripts to generate error profile	Yes	No	Yes	No	Yes	No
Substitution error rates (customizable)	No	Yes	No	Yes	No	Yes (uniformly)
Indel error rates (customizable)	Yes (uniformly)	No	No	Yes	No	No
Quality score profiles (customizable)	Min/max or quality profile	Fixed score	No	Yes	No	No
Error profiles of forward and reverse reads (customizable)	Yes	Yes	No	Yes	No	No
Fragment length	customizable distribution (*default: mean 300* *SD 30*)	customizable distribution (*default: mean 500* *SD 50*)	non-customizable	customizable distribution (*default: mean 300* *SD 30*)	customizable distribution (*default: mean 300* *SD 30*)	customizable distribution (*default: mean 500* *SD 50*)
GC-bias	No	No	Yes	No	Yes	No
Variant incorporation	No	Yes	No	Yes	Yes	No
“Golden” .bam^a^	Yes	No	No	Yes	Yes	No
Multi-threading	No	No	Yes	Yes	No	No

^a^"ground truth" read alignments.

## Materials and methods

### Simulations

#### Basic models

The referenced assembly for Baker’s yeast (*Saccharomyces cereviseae*) strain s288C (sacCer3), was downloaded from NCBI GenBank (accession number: GCA_000146045.2). The genome, consisting of 16 nuclear chromosomes and the mitochondrion, exhibits a total length of 12,157,105 bp. From this reference assembly, paired-end (PE) reads were simulated at 100× coverage using six popular short-read simulators: ART v.2.5.8 (Huang et al. 2012), DWGSIM v.0.1.15 (Homer 2022), InSilicoSeq (ISS) v.1.5.4 (Gourlé et al. 2019), Mason v.2.0.9 (Holtgrewe 2010), NEAT v.3.0 (Stephens et al. 2016), and wgsim v.0.3.1-r13 (Li et al. 2009).

Out of the six short-read simulators, ISS offers one of the largest ranges of built-in platform-specific error models (Table 1). Namely, in addition to a standard error model (“*--mode basic*”) that utilizes a kernel density estimator for generating (125 bp PE) reads, ISS contains a set of pre-computed error models for commonly used Illumina sequencers: HiSeq (126 bp PE), NovaSeq (151 bp PE), and MiSeq (301 bp PE) (Gourlé et al. 2019). ISS “*generate*” was used to simulate reads (“*-n_reads* ReadCount”) from the sacCer3 reference assembly (“*--genomes*”) under each error model (“*--model*”) with and without GC-bias (“*--gc_bias*”) using an abundance file (“*--abundance_file*’) to sample reads proportional to the length of each chromosome. For comparison, reads with the same lengths (i.e., 126, 151, and 301 bp) and a fragment size distribution with a standard deviation of 30 were also simulated using DWGSIM (“*dwgsim -1* ReadLength *-2* ReadLength *-d* (ReadLength * 3) *-s* 30 *-N* ReadCount -y 0 *-r* 0”), Mason (“*mason_simulator -ir* sacCer.fa *-n* ReadCount *--illumina --read-length* ReadLength *--fragment-mean-size* ReadLength * 3 *--fragment-size-std-dev* 30’), NEAT (“*gen_reads.py -r* sacCer.fa *-R* ReadLength *--pe* (ReadLength * 3) 30 *-c* Coverage”), and wgsim (“*wgsim -1* ReadLength *-2* ReadLength *-d* (ReadLength * 3) *-s* 30 *-N* ReadCount *-r* 0”). In contrast to DWGSIM, Mason, NEAT, and wgsim which are capable of simulating reads of variable length, ART (like ISS) is limited to a set of built-in models (Table 1). Thus, reads with similar lengths (125, 150, and 250 bp) and a fragment size distribution with a standard deviation of 30 were simulated using ART (“*art_illumina -i* sacCer.fa *--ss* IlluminaPlatform *-l* ReadLength *-m* (ReadLength * 3) *-s* 30”) using the following Illumina platform pre-sets: HiSeq 2500 [HS25] (125 and 150 bp), HiSeqX PCR-free [HSXn] (150 bp), HiSeqX TruSeq [HSXt] (150 bp), MiSeq v.1 [MSv1] (250 bp), MiSeq v.3 [MSv3] (250 bp).

As the number of simulated reads (output) differed from the expected number (input) for three out of the six software packages (Supplementary Table S1), reads were simulated at a coverage higher than the desired 100×, mapped to the reference using BWA-MEM v.0.7.17 (with the “ *-M*” option to mark secondary hits) (Li 2013), and then down-sampled using an in-house script (finite_downsampler.py).

#### Advanced models

Complementing the built-in basic models, several tools are capable of creating advanced models that allow users to mimic the characteristics of their genomic datasets (Table 1). To evaluate the performance of simulators under these “more realistic” scenarios, a barcoded genome-scale library of *S. cerevisiae* previously sequenced on an Illumina NovaSeq 6000 (Arita et al. 2021) was downloaded from NCBI (accession number: SRR12684926), the 150 bp PE reads mapped to the reference using BWA-MEM v.0.7.17, and down-sampled to 100× coverage. Next, two custom advanced models were built from this dataset. First, a custom sequence error model was built from the sample using ISS (“*iss model -b* Sample.bam *-o* Model”) and then used to simulate 10 million 151 PE reads (“*--model* Model.npz”). Second, empirical distributions of fragment lengths (compute_fraglen.py) and GC-coverage bias (compute_gc.py) were calculated from the sample using NEAT and a custom sequence error model (genSeqErrorModel.py) built. Using these features from the real data, 151 bp PE reads were then simulated to a coverage of 240X (“*gen_reads.py -r* sacCer.fa *-R* ReadLength *--pe -c* Coverage *--pe-model* FragmentLength.p *--gc-model* GCBias.p *-e* SequenceErrorModel.p”). In both cases, simulated reads were mapped back to the reference and down-sampled to 100× coverage prior to calculating any summary statistics (see “Analyses”).

### Analyses

To evaluate the performance of each read simulator, several tests were conducted:

To assess whether simulated reads were sampled uniformly across the genome, the proportion of reads mapping to each chromosome was determined (Supplementary Fig. S1). Coverage was calculated for each site in the genome using SAMtools depth v.1.9 (with the “*-a*” flag to include sites with no coverage) (Li et al. 2009) and 1.2 million sites (~10% of the genome) were randomly sampled 50 times to obtain means and standard deviations (Supplementary Fig. S2). In addition, the coverage of the first 2 kb of each chromosome was plotted using R v.4.0.2 (R Core Team 2021) (Supplementary Fig. S3).

**Fig. 1:**
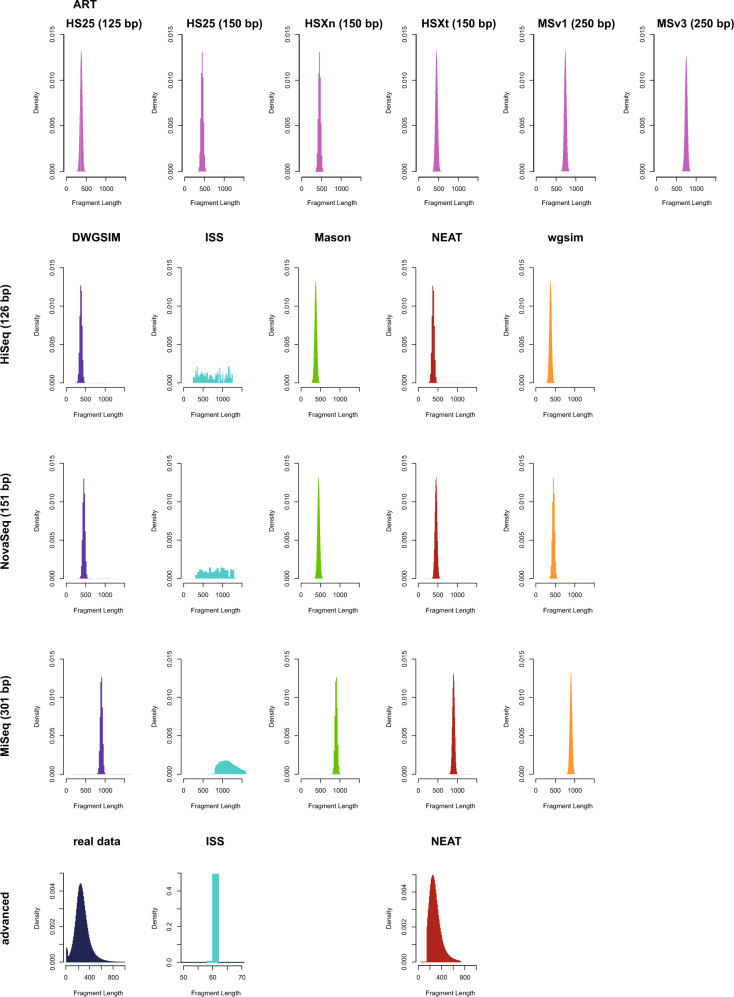
Fragment length distribution of simulated reads. Fragment length distributions for reads simulated using ART (pink) under the HS25–125bp, HS25–150bp, HSXn-150bp, HSXt-150bp, MSv1–250bp, and MSv3–250bp models as well as DWGSIM (purple), ISS (teal), Mason (green), NEAT (red), and wgsim (orange) under each basic model (HiSeq-126 bp, NovaSeq-151 bp, and MiSeq-301 bp) and under the custom advanced models containing the sequence error models built from the real data (ISS and NEAT).

To assess whether simulated reads exhibit fragment lengths similar to those expected from genuine Illumina sequencing data, paired-end fragment length distributions were calculated using an in-house script (fraglength_dist.py) and plotted using R v.4.0.2 (Fig. 1).

To assess alignment quality, read mapping statistics were calculated using SAMtools flagstat v.1.9 (Supplementary Table S2). In addition, ART, Mason, and NEAT can generate a “golden” (ground truth) set of aligned reads that indicates the regions sampled from the reference assembly which allowed for the calculation of substitution, insertion, and deletion rates and associated quality scores from the simulated data using an in-house script (find_sequencing_errors.py) (Supplementary Table S3; Supplementary Figs. S4 and S5). Overall quality scores for reads were calculated with FastQC v.0.11.7 (Andrews 2010) and plotted using MultiQC v.1.13.dev0 (Ewels et al. 2016) (Supplementary Fig. S6).

**Fig. 2:**
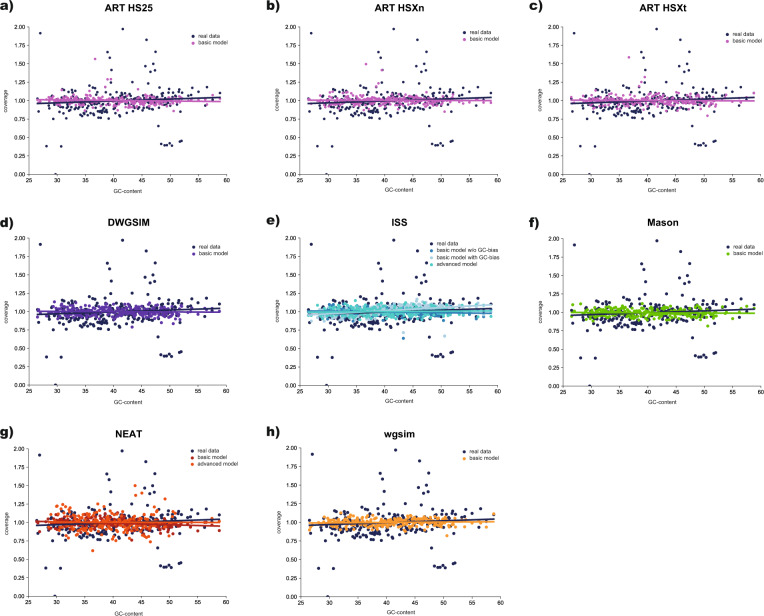
GC-bias of simulated reads. GC-bias observed in the real data (dark blue) as well as in reads simulated using ART (pink) under the HS25–150bp, HSXn-150bp, and HSXt-150bp models as well as DWGSIM (purple), ISS (teal), Mason (green), NEAT (red), and wgsim (orange) under the basic NovaSeq (151 bp) model (all software packages) with and without GC-bias (ISS only) and under the custom advanced models built from the real data (ISS and NEAT).

To assess whether simulated reads faithfully mimic the GC-coverage bias observed in real Illumina data, the GC content of the reference genome was calculated in non-overlapping 1 kb windows, compared against the scaled depth of coverage (i.e., the depth of a window divided by the average genome depth) of the simulated data, and a linear line of best fit plotted using R v.4.0.2 (Fig. 2).

**Fig. 3:**
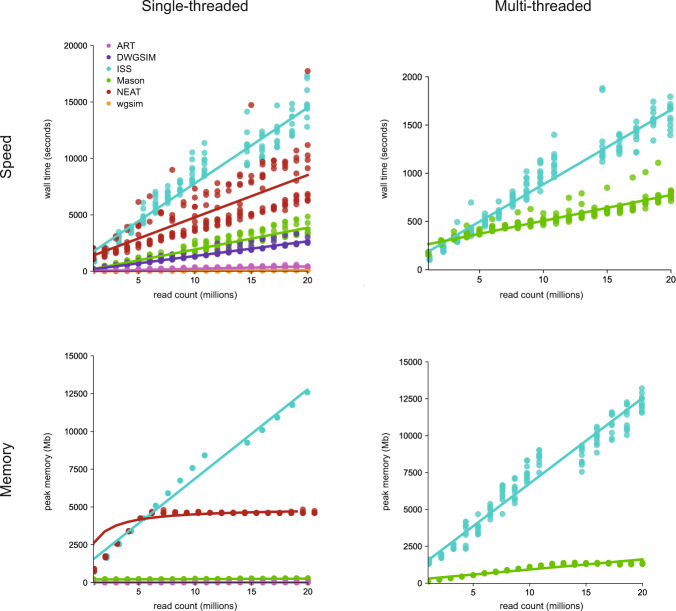
Benchmarking of read simulations. Single- and multi-threaded benchmarks for read simulations using ART (pink) under the HS25–125bp model as well as DWGSIM (purple) ISS (teal), Mason (green), NEAT (red), and wgsim (orange) under the basic HiSeq-126bp model performed on identical Broadwell CPU nodes. Wall clock time and peak memory were determined using the Unix “time” command and the efficiency script (“seff”) built-in the SLURM workload manager (Yoo et al. 2003), respectively.

To evaluate computational costs, single- and (if available) multi-threaded benchmarks (*n* = 12) were performed on identical Intel® Xeon® CPU E5-2680 v4 @ 2.40 GHz nodes. Specifically, wall clock time and peak memory were determined using the Unix “time” command and the efficiency script (“seff”) built-in the SLURM workload manager (Yoo et al. 2003), respectively (Fig. 3).

## Results

The performance of six popular short-read simulators was assessed by comparing several characteristics of the generated synthetic reads with those of an empirical Illumina sequencing dataset containing a barcoded genome-scale library of *S. cerevisiae*. For this purpose, reads were simulated with DWGSIM, ISS, Mason, NEAT, and wgsim under three basic models with and without GC-bias (ISS only) using read lengths of 126 bp (HiSeq), 151 bp (NovaSeq), and 301 bp (MiSeq) as well as under a custom advanced model built from the real data (ISS and NEAT). In addition, reads were simulated with ART under five built-in Illumina platform models of similar read length: HiSeq 2500 (125 and 150 bp), HiSeqX PCR-free (150 bp), HiSeqX TruSeq (150 bp), MiSeq v.1 (250 bp), MiSeq v.3 (250 bp).

Out of the six software packages, only three (ART, DWGSIM, and Mason) simulated the exact number of reads requested whereas ISS, NEAT, and wgsim over- or under-sampled reads (Supplementary Table S1). To allow for fair comparisons between the tools, reads were thus simulated at a higher than desired coverage and subsequently down-sampled to 100×. Thereby, ART simulated an equal number of paired-end reads per chromosome whereas all other methods sampled reads uniformly across the genome (Supplementary Fig. S1), with genomic coverages averaging close to the expected 100× (with the exception of the ART simulation under the MiSeq v.3 and ISS simulation under the basic MiSeq-301bp model which exhibit lower coverages; Supplementary Fig. S2). Reduced coverage was observed in the telomeric regions of the chromosomes, with longer read lengths resulting in higher rates of reduction (Supplementary Fig. S3).

The fragment lengths of the paired-end reads simulated under the three basic models in DWGSIM, Mason, NEAT, and wgsim as well as under the five built-in Illumina platform models in ART exhibited narrow, symmetric distributions whereas those obtained from ISS were more broadly distributed (Fig. 1). Under the custom advanced models, the fragment length distribution of reads simulated with NEAT closely resembled that of the real data (with the exception of the left tail) whereas reads simulated with ISS exhibited fragment lengths that were distinctly different from those observed in the Illumina dataset (Fig. 1).

Independent of the read simulator, all synthetic reads generated under the basic models were successfully mapped to the reference assembly, however, 3.5% of the read pairs generated in ISS using the standard error model were incorrectly split onto different chromosomes (Supplementary Table S2). In contrast to DWGSIM, ISS, and wgsim for which the regions sampled from the reference assembly remain unknown, ART, Mason, and NEAT output a “ground truth” set of aligned reads (a so-called “golden”.bam file) which demonstrated that between 97.1 and 98.3% of reads were correctly mapped back to their original positions (with the exception of the ART simulation under the MiSeq v.3 for which only 87.6% of reads were correctly mapped back; Supplementary Table S2). On average, these reads contain substitutions, insertions, and deletions errors at rates of 4.1 × 10^−3^, 4.9 × 10^−5^, and 4.8 × 10^−5^ per base in Mason and 7.0 × 10^−3^, 4.3 × 10^−5^, and 4.8 × 10^−5^ per base in NEAT, respectively (Supplementary Table S3). In contrast to Mason and NEAT, ART’s substitution, insertion, and deletion error rates depend on the Illumina platform model, with substitution rates ranging from 1.0 × 10^−3^ (MiSeq v.3) to 9.7 × 10^−4^ (HiSeq 2500–150 bp) per base, insertion rates ranging from 4.8 × 10^−7^ (HiSeq 2500–125 bp) to 9.6 × 10^−7^ (MiSeq v.1) per base, and deletion rates ranging from 1.0 × 10^−6^ (HiSeq 2500–125 bp) to 2.0 × 10^−6^ (MiSeq v.1 and v.3) per base. In NEAT, all types of errors increase towards the end of the read whereas insertion and deletion errors in Mason are independent of the base position within the read (Supplementary Fig. S4). In ART, substitution, insertion, and deletion errors under the HiSeq 2500–125 bp, HiSeqX PCR-free, HiSeqX TruSeq, and MiSeq v.1 models follow a similar trend than that observed in Mason, whereas substitution errors are elevated at the beginning of the read under the MiSeq v.3 model and remain largely constant under the HiSeq 2500–150 bp model. In concordance, quality scores, which measure the probability of an erroneous base call (Supplementary Fig. S5), decrease with increasing read length in most simulations (Supplementary Fig. S6). Exceptions include simulations using DWGSIM and wgsim as well as the custom advanced models in ISS and NEAT for which scores are reported to be of consistently poor (<20; DWGSIM and wgsim) or high (>35; ISS and NEAT) quality. Moreover, synthetic reads generally mimic the GC-coverage bias observed in the empirical Illumina data well, with the closest matches being observed under the custom advanced models (Fig. 2).

Benchmarking of computational costs highlighted that ART and wgsim outperformed all other methods under single-threaded conditions whereas Mason outperformed ISS under multi-threaded conditions (Fig. 3).

## Discussion

A faithful representation of empirical data is of great importance when using synthetic datasets to evaluate the performance of, and characterize the uncertainty in, computational pipelines. Read mapping statistics highlighted that synthetic reads closely resembled the distinct genomic regions of the reference assembly that they were simulated from. The mapping of these short reads can be complicated by repeat structures in the genome, such as the simple sequence repeats enriched in the telomeres of many species, which often results in reduced coverage in these regions in real applications (Li and Freudenberg 2014)—a pattern that is faithfully replicated, with increasing synthetic read length resulting in larger “edge effects”.

Although nearly all software packages sampled reads uniformly across the genome, three of the six tested tools over- or under-sampled reads with respect to the requested read number/coverage—a behavior that may pose issues in certain applications, for example when benchmarking the effect of genomic coverage on variant calling. Complicating the issue even further, neither the standard kernel density estimator model (Basic), the built-in platform-specific error models (HiSeq-126bp, NovaSeq-151bp, and MiSeq-301), nor the custom advanced model in ISS mimicked the fragment length distribution of the Illumina dataset well.

The probability of an error (*P*_*err*_) is directly related to the base quality score (*q*) emitted by the sequencer (*P*_*err*_ = 10^(−*q*/10)^), with error profiles varying by sequencing technology (Dohm et al. 2008; Nakamura et al. 2011). In Illumina sequencing, substitution errors increase (and hence quality scores decrease) as a function of the base position in the read due to reduced signal intensity (Kircher et al. 2009). In contrast, insertion and deletion errors, which occur at a much lower rate than substitutions (in the order of 10^−6^ compared to 10^−3^ errors per base), tend to be more evenly distributed across the length of the read (Schirmer et al. 2016). Importantly though, error rates are generally not equal between two reads in a pair, with error rates in forward reads being often much lower (up to half) than those observed in reverse reads (Schirmer et al. 2016). In addition, error rates depend on the fragment length, with higher error rates in longer fragments (Tan et al. 2019). Although substitution error rates of the synthetic reads from all simulators closely resemble those expected from Illumina data (at 10^−3^ errors per base), insertion and deletion error rates are an order of magnitude lower in ART (10^−7^) and higher in Mason and NEAT (10^−5^) than expected. At the same time, only the error profiles of Mason (all simulated models) and ART (simulations under the HiSeq 2500–125 bp, HiSeqX PCR-free, HiSeqX TruSeq, and MiSeq v.1 models) mimic both the increase in substitution rate towards the end of the reads as well as the relatively constant rate of insertions and deletions. As anticipated, most substitution errors in ART and NEAT occur at sites with low base quality scores (≤15) but the majority of insertion errors exhibit medium to high-quality scores (>20). In contrast, both substitution and insertion error rates are elevated at high-quality scores (around 40) in Mason. It should further be noted that neither differences in error rates between forward and reverse reads nor differences due to fragment length are implemented/observed in any of the tested models.

Despite the fact that differences in library preparation protocols are known to impact the rates and patterns of errors (Acinas et al. 2005), only ART contains implementations for different library designs (i.e., PCR-free and TruSeq HiSeqX models). Importantly, PCR amplification can introduce GC-coverage bias (Sims et al. 2014), with higher GC content generally leading to increased sequencing coverage (Dohm et al. 2008). Similar to systematic errors, the extent of GC-coverage bias also depends on the sequencing platform (Ross et al. 2013). Independent of the model, all methods faithfully emulated the bias observed in the real Illumina dataset.

Taken together, despite being based on a different (outdated) Illumina platform (the Genome Analyzer first launched in 2006) and with no option to implement custom advanced models, Mason accurately mimics most characteristic features of modern Illumina platforms. With the exception of insertion and deletion error profiles, NEAT too resembles empirical Illumina data well, though it is considerably slower than Mason and does not currently offer multi-threading. Of the single-threaded software, ART and wgsim outperform all other tools with regards to computational costs. Importantly, ART, Mason, and NEAT provide insights into the “ground truth”—a feature indispensable for reliable benchmarking. In contrast, fragment length distributions of both basic and advanced models simulated with ISS as well as quality scores of reads simulated with DWGSIM and wgsim were a poor representation of the empirical data tested in this study. Moreover, the lack of a “ground truth” unfortunately excludes their usage in many benchmarking and performance comparisons.

## Conclusion

In closing, it is important to keep in mind that empirical data is often highly complex, thus synthetic reads will inevitably present a simplification that may miss key components of the studied data. Yet, although there is no single-best short-read simulator, tools can be selected such that the characteristic of interest—be it genomic coverage, distribution of fragment lengths, quality scores, and systematic errors, or GC-coverage bias—will be faithfully represented (see Table 2 for an overview of the strengths and weaknesses of each software). Moreover, as tools employ different strategies, the use of multiple simulators will be highly advantageous in many benchmarking scenarios. Moreover, future implementations will likely address several of the current short-comings by implementing new features—however, whenever possible, we recommend that evaluations should ideally be based on a combination of synthetic and empirical “gold-standard” data. With the ever-decreasing costs of high-throughput sequencing, the latter will hopefully soon become available for non-model organisms.

**Table 2:** Overview of strengths (+) and weaknesses (−) of each simulator (with o being neutral).

Characteristic	ART	DWGSIM	ISS	Mason	NEAT	wgsim
Accurate read count	+	+	– (over-sampled)	+	– (over-sampled)	**o** (slightly under-sampled)
Uniform sampling across the genome	– (sampled equal # reads per chromosome)	+	+	+	+	+
Genomic coverage	**o** (– MiSeq v.3; + all other models)	+	**o** (– MiSeq model; + all other models)	+	+	+
Edge effects	+	+	+	+	+	+
Fragment length	+	+	–	+	+	+
Mapping	+	+	**o**	+	+	+
“Golden” .bam^a^	+	–	–	+	+	–
Substitution rates (10^−3^ per base)	**o** (depending on model)	^b^	^b^	+	+	^b^
Indel rates (10^−6^ per base)	– (lower at 10^−7^)	^b^	^b^	– (higher at 10^−5^)	– (higher at 10^−5^)	^b^
Substitution error profile	+	^b^	^b^	– (errors exhibit high base quality scores)	+	^b^
Indel error profile	**o** (– HiSeq2500 150 bp; – MiSeq v.3; + all other models)	^b^	^b^	– (errors exhibit high base quality scores)	– (rates increase with base position)	^b^
Forward and reverse read error rates	–	^b^	^b^	–	–	^b^
Quality profile	+	– (reports consistently poor quality scores)	**o** (advanced model reports consistently high quality scores)	+	**o** (advanced model reports consistently high-quality scores)	– (reports consistently poor-quality scores)
GC-bias	+	+	+	+	+	+
Runtime benchmark ranking – Single-threaded (1 being fastest)	**2**	**3**	**6**	**4**	**5**	**1**
Memory benchmark ranking – Single-threaded (1 needing the least memory)	**1**	**1**	**6**	**1**	**5**	**1**
Runtime benchmark ranking – Multi-threaded (1 being fastest)	**N/A**	**N/A**	**2**	**1**	**N/A**	**N/A**
Memory benchmark ranking – Multi-threaded (1 needing the least memory)	**N/A**	**N/A**	**2**	**1**	**N/A**	**N/A**

^a^"ground truth" read alignments.

^b^no "golden" .bam available for testing.

## Supplementary information

Supplementary Tables

Supplementary Figures

## Data Availability

In-house scripts are available on GitHub: https://github.com/PfeiferLab/simulator_comparison.
