# Supplementary material for: Performance evaluation of six popular short-read simulators
Source: Heredity (Edinb). 2022 Dec 10;130(2):55–63. doi: 10.1038/s41437-022-00577-3 (PMC9905089; doi:10.1038/s41437-022-00577-3)
Supplement: Supplementary file 1 — Supplementary Tables [file 41437_2022_577_MOESM1_ESM.docx]

**Supplementary Table S1**. Basic models.

| **Simulator** | **Error model** | **Read length** | **input** | **# reads (expected)** | **coverage (expected)** | **# reads (observed)** | **coverage (observed)** | **# reads  (down-sampled)** | **coverage  (down-sampled)** |
| --- | --- | --- | --- | --- | --- | --- | --- | --- | --- |
| ART | HS25  HS25  HSXn  HSXt  MSv1  MSv3 | 125 bp  150 bp  150 bp  150 bp  250 bp  250 bp | 1,500,000^1^  1,500,000^1^  1,500,000^1^  1,500,000^1^  1,500,000^1^  1,500,000^1^ | 48,000,000  48,000,000  48,000,000  48,000,000  48,000,000  48,000,000 | 497X  596X  596X  596X  994X  994X | 48,000,000  48,000,000  48,000,000  48,000,000  48,000,000  48,000,000 | 497X  596X  596X  596X  994X  982X | 9,657,060  8,047,551  8,047,551  8,047,551  4,828,528  4,828,528 | 100X  100X  100X  100X  100X  100X |
| DWGSIM |  | 126 bp  151 bp  301 bp | 6,000,000^2^  5,000,000^2^  5,000,000^2^ | 12,000,000  10,000,000  10,000,000 | 125X  150X  249X | 12,000,000  10,000,000  10,000,000 | 125X  150X  249X | 9,580,417  7,994,256  4,010,407 | 100X  100X  100X |
| ISS | Basic | 125 bp | 10,000,000^3^ | 10,000,000 | 104X | 10,852,330 | 113X | 9,657,060 | 100X |
|  | HiSeq | 126 bp | 10,000,000^3^ | 10,000,000 | 104X | 10,852,330 | 113X | 9,580,417 | 100X |
|  | NovaSeq | 151 bp | 10,000,000^3^ | 10,000,000 | 125X | 10,852,330 | 136X | 7,994,256 | 100X |
|  | MiSeq | 301 bp | 10,000,000^3^ | 10,000,000 | 249X | 10,852,332 | 270X | 4,010,407 | 100X |
| Mason |  | 126 bp | 5,000,000^2^ | 10,000,000 | 104X | 10,000,000 | 104X | 9,580,417 | 100X |
|  |  | 151 bp | 5,000,000^2^ | 10,000,000 | 125X | 10,000,000 | 125X | 7,994,256 | 100X |
|  |  | 301 bp | 5,000,000^2^ | 10,000,000 | 249X | 10,000,000 | 249X | 4,010,407 | 100X |
| NEAT |  | 126 bp | 120X^4^ | 11,496,501 | 120X | 11,836,172 | 123X | 9,580,417 | 100X |
|  |  | 151 bp | 120X^4^ | 9,593,107 | 120X | 9,874,618 | 124X | 7,994,256 | 100X |
|  |  | 301 bp | 120X^4^ | 4,812,489 | 120X | 4,950,110 | 124X | 4,010,407 | 100X |
| wgsim |  | 126 bp  151 bp  301 bp | 5,000,000^2^  5,000,000^2^  5,000,000^2^ | 10,000,000  10,000,000  10,000,000 | 104X  125X  249X | 9,999,998  9,999,998  10,000,000 | 104X  125X  237X | 9,580,147  7,994,256  4,010,407 | 100X  100X  100X |

^1^ # paired-end reads per contig; ^2^ # paired-end reads; ^3^ # reads; ^4^ coverage

**Supplementary Table S2**. Read mapping statistics of the basic models.

| **Simulator** | **Error model** | **Read length** | **% Reads mapped** | **% Reads (properly paired)** | **# mates mapped to a different chromosome** | **% correctly mapped reads^1^** |
| --- | --- | --- | --- | --- | --- | --- |
| ART | HS25  HS25  HSXn  HSXt  MSv1  MSv3 | 125 bp  150 bp  150 bp  150 bp  250 bp  250 bp | 100%  100%  100%  100%  100%  100% | 100%  100%  100%  100%  100%  100% | 0  0  0  6  10  16 | 97.41%  97.62%  97.45%  97.56%  97.78%  87.58% |
| DWGSIM |  | 126 bp  151 bp  301 bp | 100%  100%  100% | 100%  100%  100% | 2  4  2 | –  –  – |
| ISS | Basic | 125 bp | 100% | 100% | 342,022 (3.5%) | – |
|  | HiSeq | 126 bp | 100% | 100% | 0 | – |
|  | NovaSeq | 151 bp | 100% | 100% | 0 | – |
|  | MiSeq | 301 bp | 100% | 100% | 1 | – |
| Mason |  | 126 bp | 100% | 100% | 0 | 97.31% |
|  |  | 151 bp | 100% | 100% | 0 | 97.49% |
|  |  | 301 bp | 100% | 100% | 0 | 98.29% |
| NEAT |  | 126 bp | 100% | 100% | 2 | 97.05% |
|  |  | 151 bp | 100% | 100% | 2 | 97.24% |
|  |  | 301 bp | 100% | 100% | 1 | 97.94% |
| wgsim |  | 126 bp  151 bp  301 bp | 100%  100%  100% | 100%  100%  100% | 0  0  4 | –  –  – |

^1^ calculated from the "golden" (ground truth) set of aligned reads (only available for ART, Mason and NEAT)

**Supplementary Table S3**. Rates of sequencing error of the basic models.

| **Simulator** | **Error model** | **Read length** | **rate of  substitution error^1^** | **rate of insertion error^1^** | **rate of deletion error^1^** |
| --- | --- | --- | --- | --- | --- |
| ART | HS25 HS25  HSXn  HSXt  MSv1  MSv3 | 125 bp  150 bp  150 bp  150 bp  250 bp  250 bp | 8.94 × 10^-4^  9.74 × 10^-4^  2.16 × 10^-3^  1.87 × 10^-3^  4.91 × 10^-3^  1.02 × 10^-3^ | 4.77 × 10^-7^  5.75 × 10^-7^  5.80 × 10^-7^  5.74 × 10^-7^  9.58 × 10^-7^  9.47 × 10^-7^ | 1.02 × 10^-6^  1.22 × 10^-6^  1.21 × 10^-6^  1.19 × 10^-6^  2.02 × 10^-6^  2.02 × 10^-6^ |
| Mason |  | 126 bp | 4.08 × 10^-3^ | 4.85 × 10^-5^ | 4.80 × 10^-5^ |
|  |  | 151 bp | 4.07 × 10^-3^ | 4.94 × 10^-5^ | 4.81 × 10^-5^ |
|  |  | 301 bp | 4.03 × 10^-3^ | 4.97 × 10^-5^ | 4.89 × 10^-5^ |
| NEAT |  | 126 bp | 7.03 × 10^-3^ | 4.27 × 10^-5^ | 4.74 × 10^-5^ |
|  |  | 151 bp | 7.02 × 10^-3^ | 4.34 × 10^-5^ | 4.76 × 10^-5^ |
|  |  | 301 bp | 7.00 × 10^-3^ | 4.24 × 10^-5^ | 4.99 × 10^-5^ |

^1^ calculated from the "golden" (ground truth) set of aligned reads (only available for ART, Mason and NEAT)
