# Supplementary material for: Performance evaluation of six popular short-read simulators
Source: Heredity (Edinb). 2022 Dec 10;130(2):55–63. doi: 10.1038/s41437-022-00577-3 (PMC9905089; doi:10.1038/s41437-022-00577-3)
Supplement: Supplementary file 2 — Supplementary Figures [file 41437_2022_577_MOESM2_ESM.docx]

**Supplementary Figure S1.** Proportion of reads on each chromosome simulated using ART (pink) under the HS25-125bp model as well as DWGSIM (purple), ISS (teal), Mason (green), NEAT (red), and wgsim (orange) under the basic HiSeq-126bp model. For comparison, chromosome length is shown in black. ART simulates an equal number of reads per chromosome. In contrast, DWGSIM, ISS, Mason, NEAT, and wgsim sample reads proportional to the chromosome length.

**Supplementary Figure S2.** Genomic coverage for reads simulated using ART (pink) under the HS25-125bp, HS25-150bp, HSXn-150bp, HSXt-150bp, MSv1-250bp, and MSv3-250bp models as well as DWGSIM (purple), ISS (teal), Mason (green), NEAT (red), and wgsim (orange) under each basic model (Basic (ISS only), HiSeq-126 bp, NovaSeq-151 bp, and MiSeq-301 bp).

**Supplementary Figure S3.** Genomic coverage at distal chromosome regions (first 2 kb) for reads simulated using ART (pink) under the HS25-125bp, HS25-150bp, HSXn-150bp, HSXt-150bp, MSv1-250bp, and MSv3-250bp models as well as DWGSIM (purple), ISS (teal), Mason (green), NEAT (red), and wgsim (orange) under each basic model (HiSeq-126 bp, NovaSeq-151 bp, and MiSeq-301 bp).


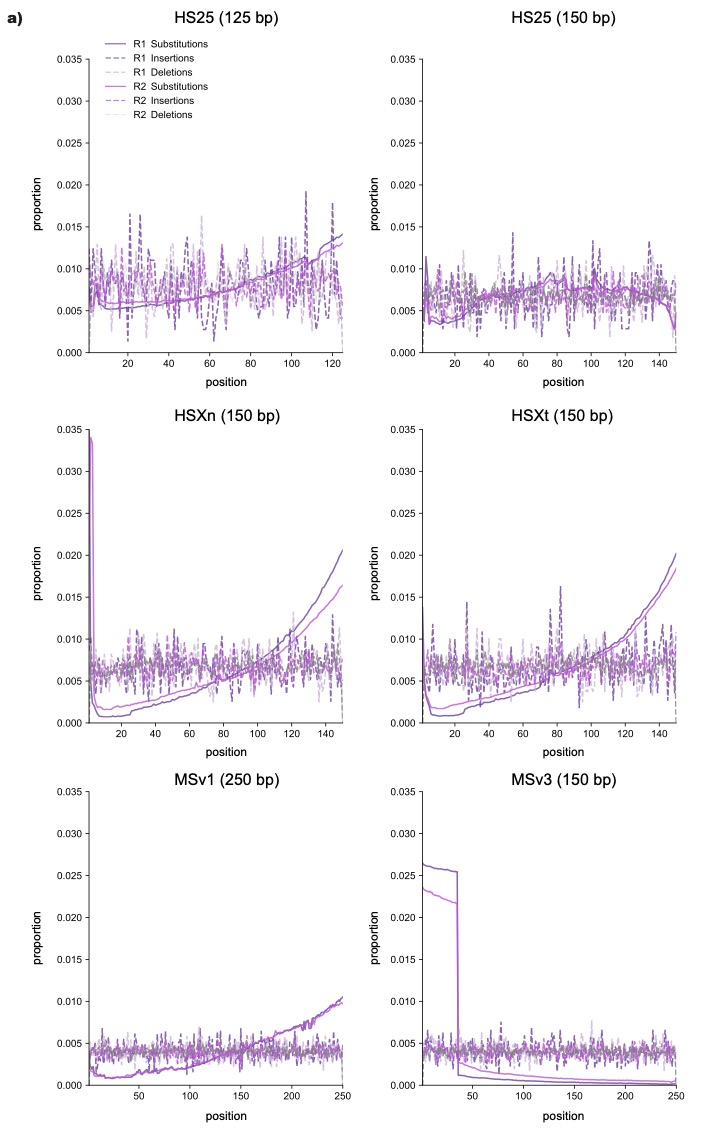


**
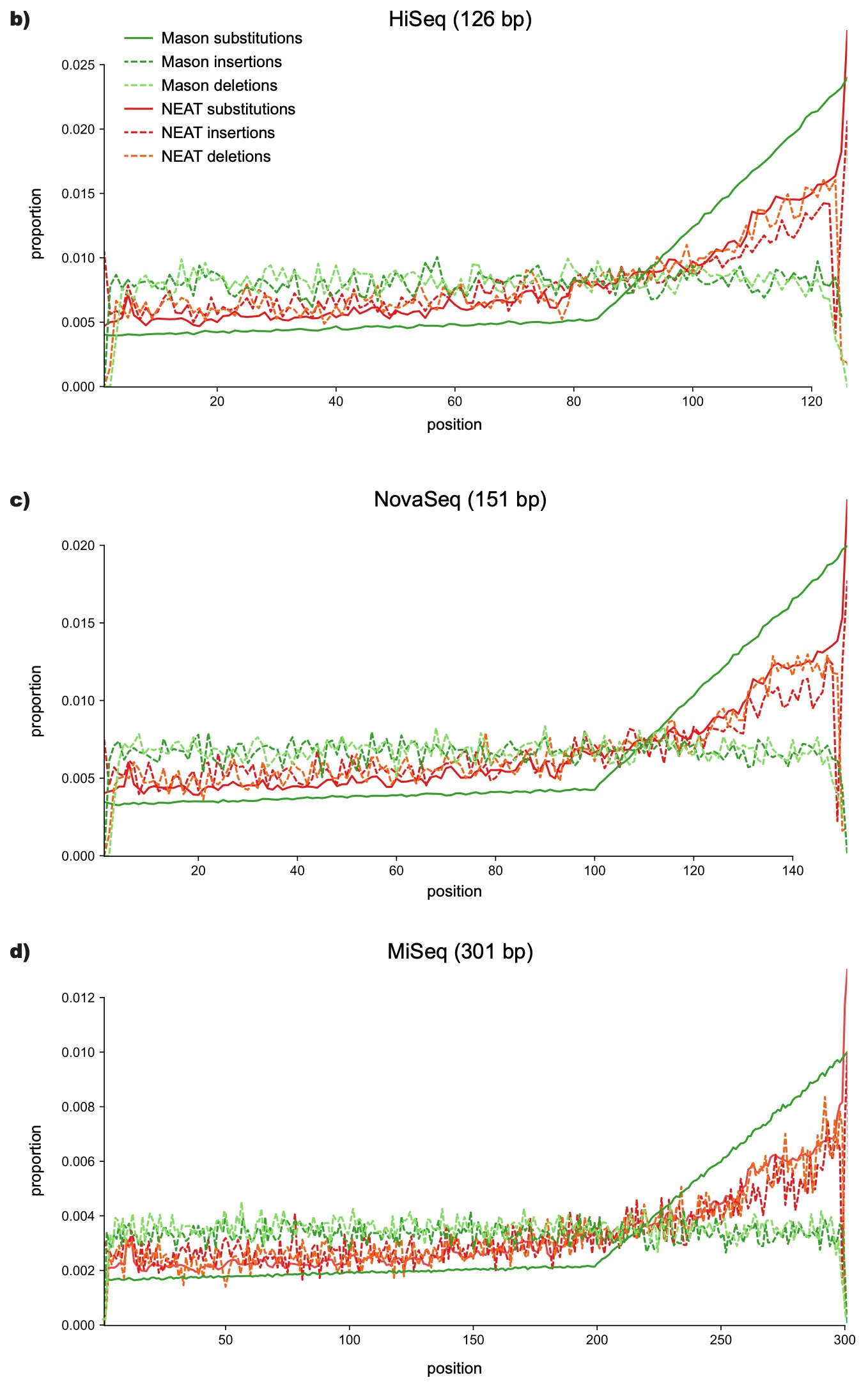
**

**Supplementary Figure S4.** Substitution, insertion, and deletion rates for reads simulated using (a) ART (pink) under the HS25-125bp, HS25-150bp, HSXn-150bp, HSXt-150bp, MSv1-250bp, and MSv3-250bp models as well as Mason (green) and NEAT (red) under each basic model ((b) HiSeq-126bp, (c) NovaSeq-151 bp, and (d) MiSeq-301bp). Calculated from the "golden" (ground truth) set of aligned reads.


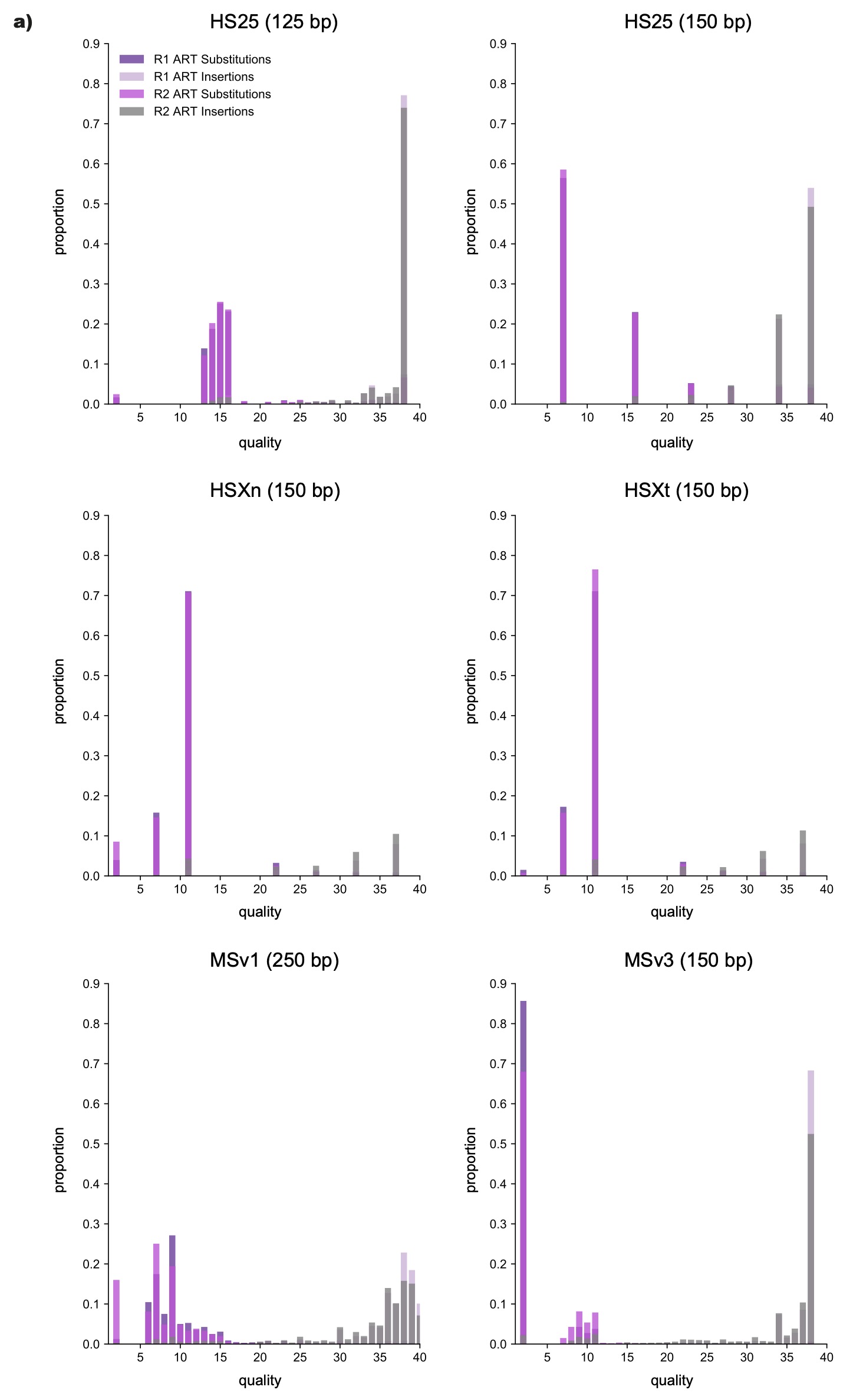


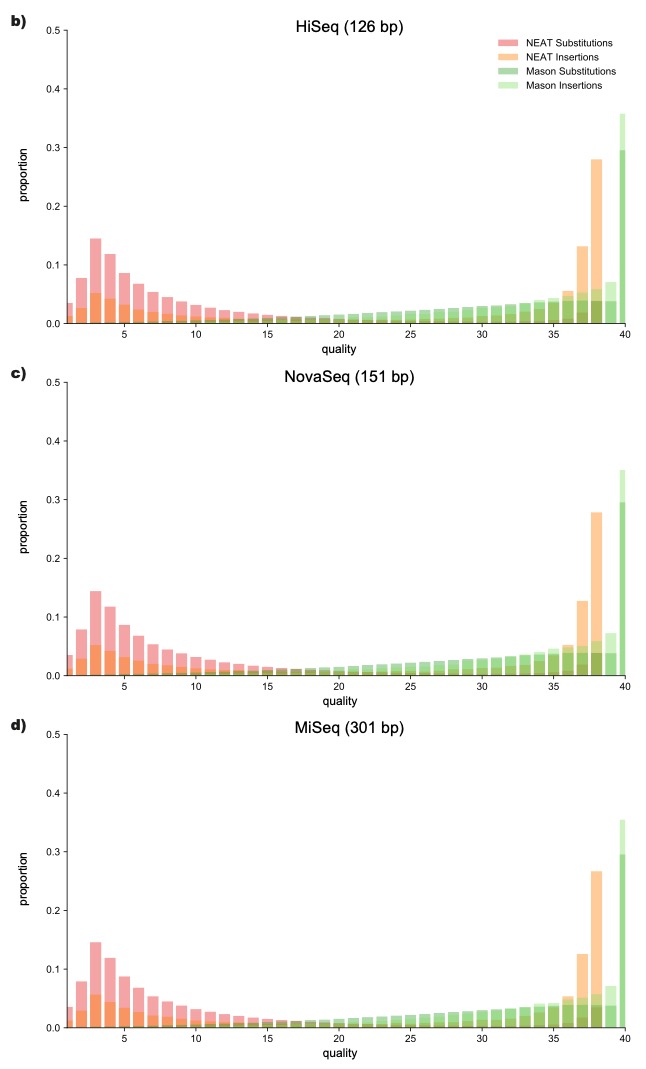


**Supplementary Figure S5.** Quality scores of substitution, insertion, and deletion errors for reads simulated using (a) ART (pink) under the HS25-125bp, HS25-150bp, HSXn-150bp, HSXt-150bp, MSv1-250bp, and MSv3-250bp models as well as Mason (green) and NEAT (red) under each basic model ((b) HiSeq-126bp, (c) NovaSeq-151 bp, and (d) MiSeq-301bp). Calculated from the "golden" (ground truth) set of aligned reads.


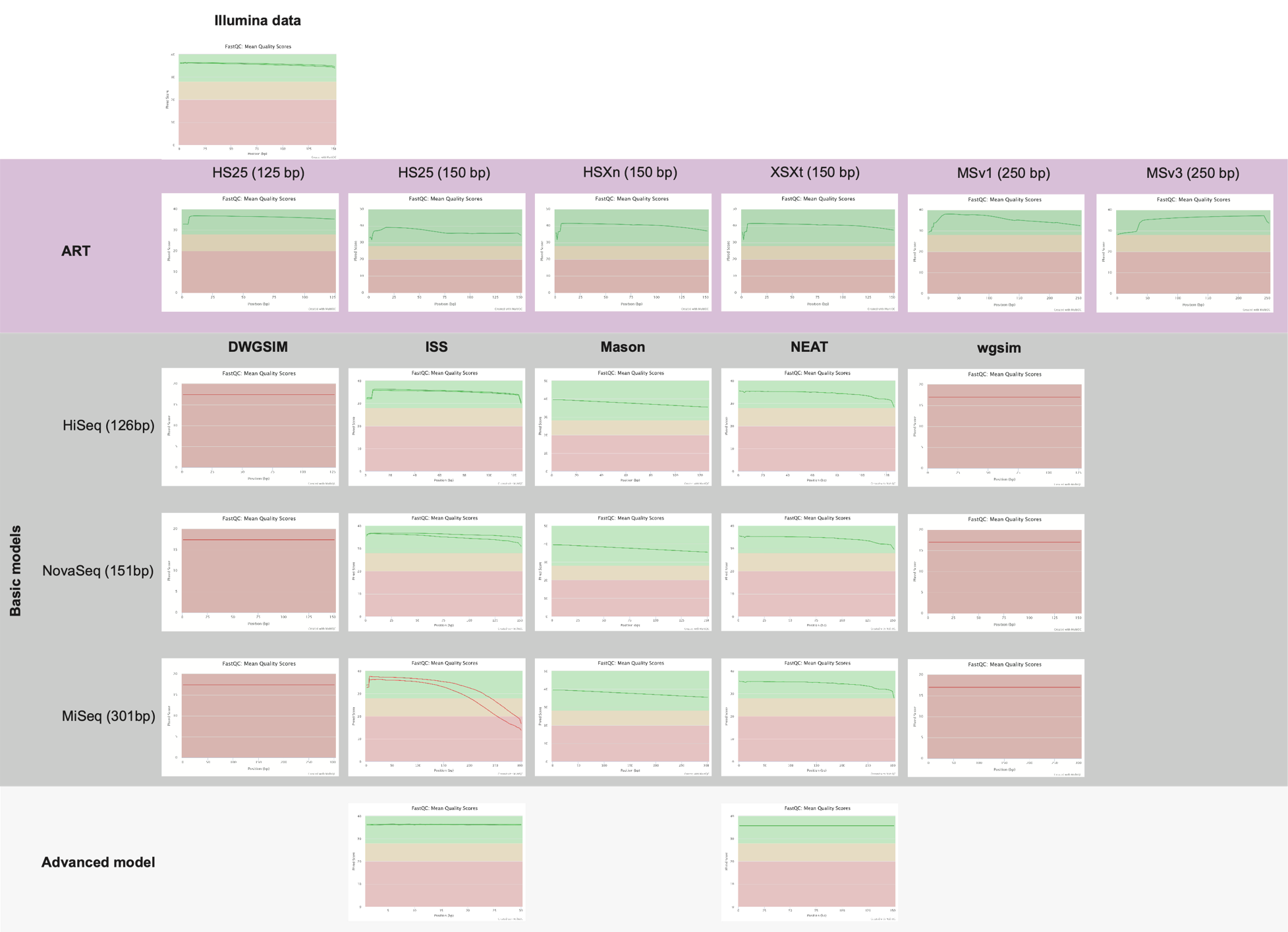


**Supplementary Figure S6.** Quality scores of reads observed in the Illumina dataset (top) as well as in reads simulated using ART under the HS25-125bp, HS25-150bp, HSXn-150bp, HSXt-150bp, MSv1-250bp, and MSv3-250bp models as well as ISS, Mason, and NEAT under each basic model (HiSeq-126bp, NovaSeq-151 bp, and MiSeq-301bp) and under the custom advanced models built from the real data (ISS and NEAT only). The x-axis indicates read position and the y-axis quality scores (green: high quality; yellow: medium quality; red: poor quality).
